# From the Editor’s Desk

**Published:** 2009-04

On this page of this issue of CVJA we publish a declaration by cardiologists in Africa, indicating strategies to combat the evergrowing burden of heart disease in Africa.

The already poor socio-economic conditions in many African countries are rapidly worsening due to rampant disease and political strife. AIDS has decimated many areas, leaving orphans to fend for themselves. Breadwinners have died, leaving their children without an income for the food necessary for normal growth and development. Lack of education because of inadequate governance undermines attempts to prevent disease. The absence of trained medical personnel leaves communities without guidance and medical support.

The incidence of rheumatic heart disease, which should have been on the decline or even eradicated, is still the commonest form of heart disease in African countries. The most susceptible are the large numbers of children in the population. Dilated cardiomyopathy of uncertain aetiology is the next most common form of heart disease, occurring in all age groups, and it is directly associated with poverty. Where developing populations come into contact with western lifestyles, the incidence of hypertension, obesity and type 2 diabetes is higher, largely related to culture shock and changing lifestyles.

For these reasons, we need a call to action to provide guidelines for strategies to combat the burden of disease in Africa. This 10-point declaration has the endorsement of all practioners of cardiology in Africa.

